# Changes in Serum Proteomic Profiles at Different Stages of Pregnancy Toxemia in Goats

**DOI:** 10.1111/jvim.70139

**Published:** 2025-06-10

**Authors:** Murat Uztimür, Cennet Nur Ünal, Gürler Akpınar

**Affiliations:** ^1^ Faculty of Veterinary Medicine, Department of Internal Medicine Bingöl University Bingol Türkiye; ^2^ Faculty of Medicine, Medical Biology Kocaeli University Kocaeli Türkiye

**Keywords:** beta‐hydroxybutyric acid, BHBA, biomarker, goat, pregnancy toxemia, proteomics

## Abstract

**Background:**

In human medicine, serum proteome profiles are used to differentiate metabolic diseases and evaluate their pathophysiology. Understanding the serum proteome profiles of goats with pregnancy toxemia might help identify the proteomes and pathways responsible for the development of this disease and improve diagnosis and treatment.

**Hypothesis/Aim:**

Determine biomarkers that differentiate healthy goats from goats with subclinical pregnancy toxemia (SPT) and clinical pregnancy toxemia (CPT) using serum proteome analysis. In addition, serum protein differences were evaluated to distinguish between SPT and CPT.

**Animals:**

Thirty‐five hair goats were included, with 15 in the SPT group, 10 in the CPT group, and 10 in the control group.

**Methods:**

The cross‐sectional study was conducted with animals from goat farms in Bingol, Türkiye, between December 2023 and May 2024. Serum samples were investigated using mass spectrometry‐based proteomic analysis.

**Results:**

Proteomic profiles showed significant variation in relative protein abundance. Twenty‐five proteins were significantly (*p* ≤ 0.01) differentially expressed between animals with pregnancy toxemia and those of the control group, with ≥ 2‐fold changes in abundance. Proteins involved in cellular, biological, and molecular processes, including processes related to reactive oxygen species, cytokine activation, acute phase response signaling, lipid metabolism, and antimicrobial activity were altered in animals with pregnancy toxemia.

**Conclusion and Clinical Importance:**

The proteomic candidates identified are biomarkers that may facilitate the diagnosis, treatment, and management of pregnancy toxemia in goats. Serum proteomic biomarkers could be used in rapid tests in the future and may improve the management of pregnancy toxemia on farms.

**Abbreviations**
ALT: alanine aminotransferase
ALT: alanine aminotransferase
ARRIVE: Animal Research: Reporting of In Vivo Experiments
BHBA: beta‐hydroxybutyric acid
CG: control group
CPT: clinical pregnancy toxemia
FDR: false discovery rate
GGT: gamma‐glutamyl transferase
HDL: high‐density lipoprotein
HPLC–MS: high‐pressure liquid chromatography–mass spectrometry
LDL: low‐density lipoprotein
MS/MS: mass spectrometry/mass spectrometry
NCE: normalized collision energy
NEB: negative energy balance
NEFA: non‐esterified fatty acid
SPT: subclinical pregnancy toxemia
TCA: trichloroacetic acid

## 1 Introduction

Pregnancy toxemia is an important metabolic disease of goats, resulting in negative energy balance as a result of impaired carbohydrate and lipid metabolism in the last trimester of pregnancy [1, 2]. Many factors, including multiple fetuses, immobility, and malnutrition, play roles in the pathogenesis of this disease [3]. Pregnancy toxemia is categorized as subclinical (SPT) or clinical pregnancy toxemia (CPT) according to clinical signs and blood beta‐hydroxybutyric acid (BHBA) concentration [1, 2]. Depending on severity, clinical signs that might be overlooked in the early stages, such as lagging behind the herd and the slowing of movements, are observed. If the disease is not treated, neurological signs, including inability to stand, leaning the head against objects, muscle tremors, bruxism, opisthotonus, and blindness, are observed [1, 2, 3]. The diagnosis of pregnancy toxemia is made by history, clinical signs, and determination of ketone bodies such as urine or serum BHBA concentration. The prevalence of pregnancy toxemia in sheep has been reported to vary between 14.86% and 20% [4] whereas it ranges between 13.3% and 23.87% in goats [5, 6, 7]. A study on the prognosis of this disease in goats reported that the survival rate after discharge from veterinary care was 70% [1]. However, another study of goats with pregnancy toxemia reported an 86% mortality rate despite cesarean delivery or induction of labor and medical treatment to correct the underlying glucose deficiency and metabolic acidosis [2]. Metabolic profile parameters, acute phase proteins, BHBA concentration, reduction–oxidation (redox) balance, blood gas parameters, and free amino acid profiles have been evaluated for diagnostic purposes in ruminants, particularly in goats with pregnancy toxemia [7, 8, 9, 10, 11, 12]. However, only a few serum biomarkers are commercially available for clinical use. The pathophysiology and progression of this disease are not fully understood.

Traditional biomedical research has focused on the analysis of single genes, proteins, metabolites, or metabolic pathways in diseases. This molecular reductionist approach is based on the assumption that identifying genetic variations and molecular components will lead to new treatments for diseases [13, 14, 15, 16]. However, many diseases are complex and multifactorial, and in order to determine the phenotype of such diseases, it is necessary to understand the changes that occur in more than one gene, pathway, protein, or metabolite at the cellular, tissue, and organismal levels [17, 18, 19]. Therefore, in recent years, proteomics, as one field of multi‐omics technologies, has helped in evaluating the complex pathogenetic mechanisms of different diseases from a broad perspective and has made substantial contributions [20, 21]. In veterinary medicine, proteomic analysis of metabolic diseases such as ketosis [16], hypocalcemia [22], and fatty liver [23] in dairy cows has contributed valuable insights for the definition of new pathophysiological pathways and new diagnosis and treatment protocols for these diseases. The proteomic approach can contribute importantly to a broad and detailed understanding of the changes that occur at the organismal level associated with the increase in BHBA concentration in goats with pregnancy toxemia. Our aim was to evaluate the serum protein profiles of goats with SPT or CPT using proteomic techniques to determine the proteomic profiles of these animals and to identify the relevant pathophysiological mechanisms.

## 2 Materials and Methods

The experimental design of this study was approved by the Bingöl University Animal Experiments Local Ethics Committee (B.Ü. HADYEK, Date: 2023/06, Decision No: 06/01) before the research began. All procedures were performed in accordance with the relevant guidelines and regulations and in accordance with the Animal Research: Reporting of In Vivo Experiments (ARRIVE) guidelines.

### 2.1 Animals

Our study included 35 hair goats aged between 2 and 6 years, all in the last 3 weeks of pregnancy. The study was conducted on a 500‐head goat farm. The studied goats had a mean weight of 55 ± 1.2 kg and a mean body condition score of 2.3 ± 0.8 [24]. Although the goats with SPT included in the study had clinical signs such as anorexia and bruxism, the goats with CPT had clinical signs such as anorexia, bruxism, ataxia, blindness, incoordination, lagging behind the herd, mucous stools, and lethargy [1]. The 35 analyzed animals were divided into three groups according to BHBA concentrations, with 15 in the SPT group, 10 in the CPT group, and 10 in the control group (CG). The BHBA and non‐esterified fatty acid (NEFA) concentrations were determined using an automated chemistry analyzer (Randox Monaco analyzer, Randox Laboratories, Crumlin, UK). The CG goats were selected from among animals with serum BHBA concentrations < 0.8 mmol/L and hematologic, biochemical, and clinical examination results (including heart rate [beats per minute] and respiratory rate [breaths per minute]) within the relevant reference ranges [1, 8]. Animals with serum BHBA concentrations of 0.8–1.6 mmol/L were analyzed as the SPT group, whereas animals with serum BHBA concentrations > 1.6 mmol/L were assigned to the CPT group [1, 25]. Goats with infectious diseases (e.g., *Theileria*, *Babesia*, and *Anaplasma*) or metabolic diseases (e.g., mastitis, metritis, laminitis, hypocalcemia, retained placenta), goats with a history of abortion, goats of different breeds, and goats that had received any medication were excluded from the study.

### 2.2 Collection of Serum Samples

Blood samples of animals with pregnancy toxemia (CPT or SPT) and CG animals were collected from the jugular vein into 8‐mL tubes without anticoagulant (BD Vacutainer tubes, BD, Plymouth, UK) according to appropriate techniques for the determination of the biochemical parameters of BHBAs, NEFAs, glucose, high‐density lipoproteins (HDLs), low‐density lipoproteins (LDLs), triglycerides, alanine aminotransferase (ALT), gamma‐glutamyl transferase (GGT), magnesium, albumin, and phosphorus. All blood collection processes were performed by the same person (M.U.). Blood samples were left at room temperature (20°C) for a maximum of 1 h to allow clotting and then centrifuged at 2100 × *g* for 15 min (HERMLE Z 36 HK centrifuge, HERMLE Labortechnik GmbH, Wehingen, Germany) and serum samples were extracted. Lipemic or hemolyzed samples were excluded. After centrifugation, the separated serum samples were distributed into 1‐mL Eppendorf tubes and stored at −80°C until used in analyses [26]. We analyzed biochemical variables (glucose, HDL, LDL, triglycerides, ALT, GGT, magnesium, albumin, and phosphorus) using an automatic biochemistry analyzer (Mindray BS‐2000m, Mindray, Nanshan, China). All biochemical variables of interest were analyzed within 2 months.

### 2.3 Preparation of Samples and Determination of Protein Concentrations

Serum samples were precipitated with the trichloroacetic acid (TCA)‐acetone protocol to remove salts and other sources of contamination and ensure that samples were suitable for the working conditions of high‐pressure liquid chromatography‐mass spectrometry (HPLC–MS). Briefly, one‐fourth of the volume of each sample was added to TCA samples and kept on ice for 10 min. After the tubes were centrifuged in a microcentrifuge at 14 000 × *g* for 5 min, the supernatant was carefully separated from the precipitated proteins. After the protein precipitates were washed with 200 μL of acetone, they were centrifuged in a microcentrifuge at 14 000 × *g* for 5 min and then discarded into the acetone phase. The protein pellet was left at room temperature for 5 min to remove the remaining acetone. The pellet was vortexed until dissolved in 50 mM ammonium bicarbonate and 0.1% formic acid. Protein concentrations were measured by Bradford assay (NanoDrop 1000, Thermo Fisher Scientific, Waltham, MA, USA) using prepared standards and necessary sample dilutions, and then, after quick freezing, the samples were stored at −80°C until use. Before the LC–MS analysis of the samples, 200 μg of protein was taken from individual samples of each experimental group, and proteins were combined in a tube to pool the samples. In this way, individual differences that could occur between samples were minimized. The LC–MS analysis was carried out using samples obtained from both the experimental group and CG.

### 2.4 Preparation of Proteins for Mass Spectrometry Analysis

Proteins were digested with trypsin to prepare them for MS. For this purpose, an in‐solution tryptic digestion kit (#89895, Thermo Fisher Scientific, USA) was used and the kit's protocol was followed, entailing reduction with dithiothreitol at 95°C for 5 min, alkalization with iodacetamide at room temperature in the dark for 20 min, and trypsin digestion at 37°C for 5 h or at 30°C overnight. The concentration of the obtained peptides was evaluated using a Qubit 4.0 device (Thermo Fisher Scientific).

### 2.5 LC–MS/MS Parameters

Peptide counting was performed using a Thermo Fisher Scientific Dionex Ultimate 3000 Series RSLC nanopump. This nanopump is equipped with Thermo Fisher Scientific Ultimate 3000 Series TCC‐3000RS column compartments and is connected to a Thermo Fisher Scientific Dionex UltiMate 3000 Series RS autosampling unit. The entire system was controlled using Xcalibur 4.0 software (Thermo Fisher Scientific). Before each analysis, samples were loaded onto a capture arm of 5 mm × 300 μm internal diameter, 5 μm, 100 Å, containing C18 material (Thermo Fisher Scientific), at a speed of 5 μL/min with a solution containing 0.05% (v/v) trifluoroacetic acid and 1% acetonitrile. The sample capture column then was transferred to an Acclaim PepMap RSLC analytical column (Thermo Fisher Scientific) measuring 15 cm × 75 μm and packed with C18 material of 2 μm and 100 Å, and peptide elution was performed using two different mobile phases (A and B). Mobile phase A comprised 0.1% (v/v) formic acid prepared with HPLC‐quality water, and mobile phase B comprised 0.1% (v/v) acetonitrile prepared with HPLC‐quality water. Peptides were separated for 130 min at a flow rate of 0.3 μL/min. They then were placed in a Thermo Q Exactive mass spectrometer. The column temperature was kept at 40°C, and 2 μL of sample was injected for each analysis. A heated electrospray ionization source was used to ensure the ionization of peptides in positive‐charge reading mode. The device parameters were set as follows: +2.3 kV voltage, 300°C capillary temperature, sheet gas and auxiliary gas flows of approximately 50 and 30 units, and S and RF lens levels set to 50. Samples passing through the column with continuous nanoflow were ionized and subjected to mass spectrometry/mass spectrometry (MS/MS) analysis. TOP10 MS/MS analysis was performed for each parent ion.

### 2.6 Data‐Dependent Acquisition

We used the aforementioned Q Exactive device to perform data‐dependent acquisition. The scanning process initially was performed in full MS spectrum mode with the following parameters: resolution of 70 000, scanning range of 400–200 *m/z*, 3 × 10^6^ target automatic gain control (AGC), maximum injection time of 60 ms, and centroid data type. Based on the MS results, we selected the top 10 precursor ions (TOP10) for MS/MS analysis and read these ions after high‐energy collision separation (HCD). The HCD parameters were as follows: resolution of 17 500, 1 × 10^5^ AGC, maximum injection time of 100 ms, 2 *m/z* isolation window, normalized collision energy (NCE) 27, and centroid spectrum data type for collection. Before each injection in the positive mode, we calibrated the device using the standard positive calibrant (LTQ Velos ESI Positive Ion Calibration Solution 88323, Pierce, Thermo Fisher Scientific).

### 2.7 Peptide and Protein Identification

Raw data obtained as a result of the LC–MS/MS analysis were analyzed using Proteome Discoverer 2.2 software (Thermo Fisher Scientific) for protein identification. The following parameters were used for this purpose: peptide mass tolerance of 10 ppm, MS/MS mass tolerance of 0.2 dA, mass accuracy of 2 ppm, tolerated mis‐cutting of 1, minimum peptide length of six amino acids, fixed modifications of cysteine carbamidomethylation, and unfixed modifications of methionine oxidation and asparagine deamination. The minimum number of peptides identified for each protein was accepted as 2, and data were investigated according to the organism‐specific data in the UniProt/Swiss‐Prot database. Functional and biological grouping of the proteins was done according to Gene Ontology data, which is an extension of Proteome Discoverer 2.2. Comparisons of proteins showing differences between the groups were performed with label‐free quantification. Functional and biological grouping was performed with STRING analyses.

### 2.8 Bioinformatics Analysis

Bioinformatics analyses were performed using proteins with statistically significant regulation rates among the proteins identified as a result of MS analyses. These proteins were first scanned with the STRING analysis program using their UniProt accession numbers [27]. In this process, “multiple proteins” was selected as the search, and “ *Capra hircus* ” was selected as the organism. After checking that the STRING accession numbers selected the correct proteins, the analysis parameters were adjusted. For these parameters, a medium level of confidence interaction score was selected, and the maximum number of interactions selected for shells 1 and 2 was determined to be no more than 5. High confidence was selected as 0.900. We considered data with false discovery rates (FDRs) of less than 10^−4^. Again, the UniProt accession numbers of the edited proteins were uploaded to the system, and “ *C. hircus* ” was selected as the organism. In ontological analyses, molecular function, biological process, cellular component, protein class, and cellular pathway were selected and analyzed.

### 2.9 Statistical Analysis

Data were analyzed using IBM SPSS Statistics 22.0 for Windows (IBM Corp., Armonk, NY, USA) and GraphPad Prism 9 for Windows (GraphPad Inc., San Diego, CA, USA). The Shapiro–Wilk test was used to evaluate whether the data were normally distributed. To determine the differences between the pregnancy toxemia groups and the CG, the Kruskal–Wallis test was applied, followed by the Mann–Whitney *U*‐test, or one‐way analysis of variance was applied, followed by Tukey's post hoc multiple comparisons test. The Benjamini‐Hochberg FDR *p* value correction was applied, and proteins were considered statistically significant at FDR values of < 0.05. Principal component analyses were performed using the R package ggplot2 v3.1. Statistical significance was accepted for differences between groups at *p* < 0.05 and fold changes of ≥ 2‐fold.

## 3 Results

The medians, ranges, and statistical significance levels for biochemical values of CG, SPT, and CPT goats are shown in Table 1. The BHBA concentrations in the pregnancy toxemia groups (SPT and CPT) were significantly higher than those of the CG (*p* < 0.001). The NEFA concentrations in the pregnancy toxemia groups also were found to be significantly higher compared with the CG (*p* < 0.001). The NEFA concentrations of the CPT group were significantly higher than those of the SPT group (*p* < 0.001) or the CG (p < 0.001). Additionally, the NEFA concentrations of the SPT group were significantly higher than those of the CG (*p* < 0.001). The glucose concentrations of the CPT group were significantly lower compared with CG (*p* < 0.001) and SPT (*p* < 0.002) animals. No significant difference was found between the pregnancy toxemia groups and the CG in terms of calcium (*p* > 0.10), phosphorus (*p* > 0.244), magnesium (*p* > 0.16), ALT (*p* > 0.86), LDL (*p* > 0.6), or HDL (*p* > 0.25). Triglyceride concentrations were significantly lower in the CPT (*p* < 0.01) and SPT (*p* < 0.01) groups compared with the CG. On the contrary, AST activity was significantly higher in the CPT (*p* < 0.02) and SPT (*p* < 0.04) groups compared with the CG.

**TABLE 1:** Median values, ranges, and statistical significance levels of differences between groups for biochemical values of CG, SPT, and CPT goats.

Parameters	CG	SPT	CPT	*p*
BHBA	0.4 (0.1–0.4)^a^	1.2 (0.8–1.5)^b^	3.95 (1.6–6.1)^c^	0.001
NEFA	0.5 (0.2–0.56)^a^	0.91 (0.44–1.56)^a^	0.95 (0.78–1.56)^b^	0.001
Calcium	8.4 (7.76–9.78)	8.09 (6.87–9.45)	8.4 (6.96–8.97)	0.1
Phosphorus	3.53 (2.74–4.56)	3.78 (3.16–6.59)	4.18 (2.4–7.06)	0.24
Magnesium	2 (1.67–2.74)	1.96 (1.41–2.83)	2.3 (1.84–2.69)	0.16
Glucose	55.61 (39.99–69.66)^a^	51.1 (36‐101)^a^	32.6 (20.1–59.5)^b^	0.004
ALT	14.9 (11.2–19.9)	15 (6–23)	13 (11–27)	0.86
AST	85 (64.2–132.2)^a^	101 (74‐207)^b^	118.5 (80‐259)^b^	0.04
Triglycerides	24 (8.2–37.1)^a^	16.4 (8.7–21.4)^b^	12.1 (7.6–35.3)^b^	0.001
LDL	12.6 (6.5–20.9)	14.2 (9.7–22)	8.7 (6.5–124)	0.6
HDL	42 (34–55.4)	47.9 (32.7–62.8)	50.65 (34.3–66.9)	0.25
Cholesterol	59 (45.8–82.1)	66.9 (51.1–90)	62.85 (41.3–88.9)	0.41

*Note:* Differences between groups with different superscripted letters in the same row are significant.

Abbreviations: ALT, alanine aminotransferase; AST, aspartate aminotransferase; BHBA, beta‐hydroxybutyric acid; CG, control group; CPT, clinical pregnancy toxemia; HDL, high‐density lipoprotein; LDL, low‐density lipoprotein; NEFA, non‐esterified fatty acid; SPT, subclinical pregnancy toxemia.

The down‐ or upregulated proteomes of SPT and CPT goats with significant differences compared to CG animals are shown in Table 2. Twenty‐five proteins were identified that showed significant differences in animals with pregnancy toxemia. These proteins were differentially abundant between the pregnancy toxemia groups and the CG with fold changes of ≥ 2 (*p* ≤ 0.01). Differences were detected between the pregnancy toxemia and CG as a whole, with larger differences in relative abundance observed between the SPT/CPT groups and the CG. Nineteen of these proteins (haptoglobin, apolipoprotein E, keratin, type I cytoskeletal 25, apolipoprotein C, apolipoprotein E, beta‐lactoglobulin, kappa‐casein, alpha‐S1‐casein, glycosylation‐dependent cell adhesion molecule 1, beta‐casein, cystatin‐1, keratin‐associated protein 11‐1, fibrinogen alpha chain, fibrinogen beta chain, interleukin‐1 beta, ATP synthase protein 8, chitinase‐3‐like protein 1, ATP synthase subunit a, galectin‐1) were upregulated, whereas 6 (albumin, hemoglobin subunit beta‐A, hemoglobin subunit alpha‐1, hemoglobin subunit beta‐C, inter‐alpha‐trypsin inhibitor, and lactotransferrin) were downregulated. In goats with CPT, haptoglobin (26‐fold), cystatin‐1 (12.624‐fold), apolipoprotein C (8.523‐fold), apolipoprotein E (8.623‐fold), fibrinogen alpha chain (7.162‐fold), fibrinogen beta chain (6.153‐fold), glycosylation‐dependent cell adhesion molecule 1 (6.284‐fold), beta‐lactoglobulin (2.322‐fold), and interleukin‐1 beta (2.92‐fold) were the most commonly upregulated proteins. In goats with SPT, hemoglobin (5.272‐fold), apolipoprotein C (17.692‐fold), apolipoprotein E (8.22‐fold), glycosylation‐dependent cell adhesion molecule 1 (14.502‐fold), beta‐lactoglobulin (3.611‐fold), kappa‐casein (3.959‐fold), hemoglobin subunit beta‐A (4.432‐fold), hemoglobin subunit alpha‐1 (3.269‐fold), alpha‐S1‐casein (3.335‐fold), and beta‐casein (2.115‐fold) were the most common upregulated proteins. In addition, five proteins, including cystatin‐1, haptoglobin, ATP synthase protein 8, ATP synthase subunit a, and glycosylation‐dependent cell adhesion molecule 1 showed significant differences between the SPT and CPT groups.

**TABLE 2:** List of up‐ and downregulated proteins in SPT and CPT goats with significant differences in protein expression compared to CG animals.

Accession number	Description	Abundance, CG	Abundance, CPT	Abundance, SPT	Fold change, CPT	*p*, CPT	FDR value, CPT	Fold change, SPT	*p*, SPT	FDR value, SPT	Up‐ or downregulated
B6E141	Haptoglobin	96 379 231.69	2 536 015 144	508 107 513	26.313	2.1e−10	1.4e−11	5.272	4.0e−7	1.5e−7	Upregulated
P85295	Albumin	8 823 838 079	4 766 448 408	5 507 897 922	0.54	6.4e−6	2.1e−6	0.624	1.3e−5	8.5e−6	Downregulated
P02077	Hemoglobin subunit beta‐A	343 936 285.8	619 882 866.8	1 524 386 352	1.802	4.4e−6	1.3e−6	4.432	8.9e−9	5.9e−10	Downregulated
P0CH25	Hemoglobin subunit alpha‐1	286 425 801.9	500 751 040.1	936 262 771.7	1.748	7.8e−5	3.8e−5	3.269	8.8e−7	4.1e−7	Downregulated
P0DN38	Apolipoprotein E	10 509 728.91	90 625 754.3	86 392 773.13	8.623	2.6e−6	4.3e−7	8.22	8.8e−7	4.4e−7	Upregulated
P02078	Hemoglobin subunit beta‐C	26 318 720.93	36 514 699.14	9 080 424.276	1.387	1.2e−3	7.1e−4	0.345	2.1e−6	1.3e−6	Downregulated
Q6R650	Keratin, type I cytoskeletal 25	19 858 553.56	23 316 531.02	23 834 976.93	1.174	8.9e−1	8.0e−1	1.2	1.7e−1	1.6e−1	Upregulated
P0DN40	Apolipoprotein C	1 622 421.88	13 827 129.07	28 704 568.23	8.523	6.8e−7	8.7e−8	17.692	3.4e−8	5.3e−9	Upregulated
P02756	Beta‐lactoglobulin	5 184 169.795	12 039 349.78	18 720 926.2	2.322	3.8e−6	8.4e−7	3.611	2.6e−7	8.6e−8	Upregulated
P02670	Kappa‐casein	3 632 921.45	7 267 197.84	14 381 883.59	2	2.3e−5	8.8e−6	3.959	8.0e−7	3.4e−7	Upregulated
P18626	Alpha‐S1‐casein	4 205 832.92	4 984 990.15	14 024 634.66	1.185	4.8e−1	3.9e−1	3.335	1.4e−3	9.9e−4	Upregulated
P62756	Inter‐alpha‐trypsin inhibitor	28 347 597.67	22 310 211.65	22 138 845.45	0.787	1.4e−1	1.0e−1	0.781	7.2e−2	6.3e−2	Downregulated
P01624	Immunoglobulin kappa variable 3‐15	1 243 995.737	37 616 875.48	47 955 812.77	30.239	2.1e−10	7.4e−12	38.55	4.3e−11	1.4e−12	Upregulated
Q29477	Lactotransferrin	107 868 050.1	66 069 319.09	97 920 796.91	0.613	2.5e−3	1.6e−3	0.908	2.0e−1	1.9e−1	Downregulated
P81447	Glycosylation‐dependent cell adhesion molecule 1	329 388.6072	2 069 859.662	4 776 760.545	6.284	2.4e−5	1.0e−5	14.502	2.1e−6	1.2e−6	Upregulated
P33048	Beta‐casein	12 052 790.01	13 771 334.48	25 494 794.6	1.143	7.1e−1	6.0e−1	2.115	3.0e−4	2.0e−4	Upregulated
P84911	Cystatin‐1	62 224.61047	785 508.1361	162 882.4426	12.624	1.07e−07	1.3e−06	2.618	2.5e−05	5.11e−05	Upregulated
Q6R648	Keratin‐associated protein 11‐1	1 810 703.625	25 109 178.59	50 732 982.29	13.867	6.41709e−14	3.94884e−12	28.018	6.41709e−14	2.32005e−12	Upregulated
P68215	Fibrinogen alpha chain	34 290.15625	245 601.9646	262 910.0562	7.162	3.51765e−06	1.21049e−05	7.667	3.32069e−06	9.54698e−06	Upregulated
P68117	Fibrinogen beta chain	791 830.0312	5 354 701.703	6 615 582.651	6.762	2.77583e−10	8.1193e−09	8.355	2.62745e−11	5.49377e−10	Upregulated
P79162	Interleukin‐1 beta	80 457 788.33	92299117.36	250 601 925	1.147	0.006242884	0.008440074	3.115	4.59153e−08	3.68019e−07	Upregulated
Q9MQK2	ATP synthase protein 8	14 190 736.8	32220971.15	1788169.137	2.271	0.003220686	0.004485955	0.126	2.3578e−05	4.97518e−05	Upregulated
Q8SPQ0	Chitinase‐3‐like protein 1	316 386 382.3	131 618 287.1	117 257 736.5	0.416	3.39977e−e−06	1.19268e−05	0.371	1.74382e−06	5.649e−06	Upregulated
Q32644	ATP synthase subunit a	562 502.193	1 098 886.096	20 568 418.27	1.954	1.04251e−05	2.6516e−05	36.566	4.81171e−13	1.38337e−11	Upregulated
C0HJR3	Galectin‐1	1 4300 883.81	14 261 939.26	19 518 331.67	0.997	0.76427044	0.78438282	1.365	0.0015316	0.002096833	Upregulated

Abbreviations: CG, control group; CPT, clinical pregnancy toxemia; FDR, Benjamini‐Hochberg false discovery rate; SPT, subclinical pregnancy toxemia.

The proteins that showed significant differences between the groups were found to have biological and molecular functions in areas such as transport, iron metabolism, milk proteins, protein stabilization, acute phase response, immune reaction, metabolic processes, inflammatory response, antimicrobial reaction, structural molecule activity, and multiple cellular functions in organisms (www.uniprot.org).

Among the proteins with significant expression in serum samples, beta‐lactoglobulin, glycosylation‐dependent cell adhesion molecule 1, hemoglobin subunit beta‐A, hemoglobin subunit alpha‐1, hemoglobin subunit beta‐C, ATP synthase subunit a, galectin‐1, fibrinogen beta chain, fibrinogen alpha chain, kappa‐casein, keratin‐associated protein 11‐1, chitinase‐3‐like protein 1, interleukin‐1 beta, cytochrome c oxidase subunit 1, lactotransferrin, and inter‐alpha‐trypsin inhibitor were found to have significant interactions with one another (www.string‐db.org).

## 4 Discussion

Identification of biomarkers that can provide useful and highly accurate results in the diagnosis of pregnancy toxemia in goats might enable easy and rapid diagnosis of the disease and, as a result, support effective treatment. We aimed to identify new biomarker candidates that allow the distinction between subclinical and clinical forms of pregnancy toxemia, which are commonly seen in the last trimester of pregnancy in goats. Evaluation of the effective use of these biomarker candidates in clinical practice is beyond the scope of our present study.

We determined that significant concentration changes occurred in the groups with SPT and CPT when 25 different serum proteins were compared between the animals with pregnancy toxemia and the CG. Among these proteins, haptoglobin, apolipoprotein C, inter‐alpha trypsin inhibitors, cystatin‐1, chitinase‐3‐like protein 1, and galectin‐1 showed the largest fold changes (≥ 2‐fold) compared with the animals of the CG. We focused on these proteomes because of their confirmed diagnostic, prognostic, and pathophysiological activities in different types of diseases of animal [28, 29, 30, 31, 32] as well as different diseases in humans [32, 33, 34, 35, 36, 37, 38]. In addition, because the applications of proteomics technology are very new in veterinary medicine, the information provided by our study constitutes the most current information. These proteins are involved in cellular, biological, and molecular processes such as immunoinflammatory reactions, lipid metabolism, acute phase response, and antimicrobial activity, suggesting roles for these compounds in the pathogenesis of pregnancy toxemia. In addition, the significant differences and very large fold increases in their serum concentrations in the pregnancy toxemia groups might signify the potential diagnostic value of these proteins.

Hemoglobin, an α2‐globulin, is an acute phase protein that scavenges hemoglobin during intravascular or extravascular hemolysis [39]. Previous studies have reported that haptoglobin can be a useful biomarker in goats for diagnostic and prognostic purposes in diseases such as caprine arthritis encephalitis virus [28], subclinical mastitis [29], and *Corynebacterium pseudotuberculosis* infection [39]. Similarly, in a study conducted on postpartum dairy cows, serum haptoglobin concentration was found to be a useful biomarker for diagnosing or predicting metabolic stress, ease of calving, and metritis in cows [9]. Evaluating the haptoglobin concentration in cases of uterine and metabolic diseases also has diagnostic and prognostic importance [9, 10, 40]. In our study, serum haptoglobin concentration was found to increase significantly in the presence of pregnancy toxemia, consistent with previous studies. The diagnostic or predictive power of haptoglobin in cases of uterine and metabolic diseases, such as hepatic lipidosis, should be investigated in the future in goats.

Apolipoprotein C is a protein with low molecular weight produced mainly by the liver [41]. There are no studies in the literature to date on apolipoprotein C in ruminants or carnivores, and it has only been addressed in a few studies in human medicine [33, 34, 42, 43]. Our study is the first to evaluate apolipoprotein C in goats with pregnancy toxemia. Apolipoprotein C‐II was found to be significantly increased in diabetic and prediabetic patients and was positively correlated with triglyceride concentration [33, 34, 43]. In parallel with previous studies [33, 34, 43], in our study, apolipoprotein C‐II expression was found to be increased in animals with pregnancy toxemia compared with CG animals. Apolipoprotein C‐II showed a 17.692‐fold increase in the SPT group and an 8.523‐fold increase in the CPT group. Apolipoprotein C is a potential biomarker candidate in pregnancy toxemia because of its low cost, the availability of commercially available apolipoprotein C ELISA kits, and the fact that many laboratories today provide apolipoprotein C analysis services. As shown in previous reports [33, 34, 43], apolipoprotein C‐II is important in the prediction and evaluation of patients with diabetes. Therefore, this proteome also can be used as a diagnostic and prognostic marker in animals with ketosis, which has a pathophysiology similar to that of diabetes, and in pregnancy toxemia.

Cystatin‐1 is produced in many different tissues, such as the gallbladder, submandibular gland, and uterus, and plays an important role in inflammatory tissue damage and tissue remodeling [30, 31]. Upregulation of cystatin‐1 has been shown to be associated with cell proliferation, tumor invasion, and metastasis [35]. Furthermore, the role of cystatin‐1 has been evaluated in gastric cancer, pancreatic cancer, colorectal cancer, and esophageal squamous cell carcinoma [30, 35, 36]. Therefore, it has been suggested by many researchers that this peptide could be used as a diagnostic or prognostic tumor biomarker [35, 36]. In recent years, this biomarker has been reported to be promising in studies on respiratory system diseases [44, 45] and periodontal diseases [46]. However, no study has been found in the literature evaluating the effect of cystatin‐1 on glucose and lipid metabolism. In our study, cystatin‐1 expression was found to be significantly increased in goats with pregnancy toxemia compared with animals of the CG.

Inter‐alpha trypsin inhibitors are mainly produced by the liver, whereas small amounts are also produced by monocytes, macrophages, and pulmonary alveolar cells [47]. A previous study investigated serum proteomic profiles for early diabetes diagnosis and found that serum alpha‐1 protein was diagnostic for early diabetes [48]. Similarly, another study found a decrease in serum alpha‐1 trypsin inhibitor concentration in patients with diabetes mellitus [49]. In addition to studies reporting this protein as diagnostic, some studies have evaluated its therapeutic efficacy. In a study conducted with patients with type 1 diabetes, it was reported that alpha‐1 antitrypsin use had effective, safe, and optimal therapeutic effects [50]. These positive effects of inter‐alpha trypsin inhibitors on glucose and carbohydrate metabolism confirm that they play important roles in the pathophysiology of metabolic diseases. However, these findings must be further supported by molecular and clinical studies. Consistent with the literature, we found that serum alpha‐1 protein inhibitor concentration was decreased in goats with pregnancy toxemia. The decreases in alpha‐1 protein inhibitor concentration observed in our study may be related to excessive consumption of the protein because of its immunomodulatory effects [47]. Based on these findings, serum alpha‐1 protein inhibitor might be an important negative acute phase protein in goats and a reliable biomarker for the diagnosis of pregnancy toxemia. In addition, results previously obtained using alpha‐1 protein inhibitor therapeutically in the treatment of diabetic patients indicate that it also could be used therapeutically in animals with ketosis and pregnancy toxemia. In light of these results [48, 49, 50], alpha‐1 protein inhibitor can be easily analyzed with automated systems using the nephelometric‐radioimmunoassay method and can be used in diagnostic, prognostic, and therapeutic applications in goats with pregnancy toxemia, which has a pathophysiology similar to that seen in diabetic patients [48, 49, 50].

Chitinase‐3‐like protein 1 is considered an acute phase response protein because it is produced by many different cells, such as neutrophils and macrophages, and secreted by different cells during inflammation or infection [51, 52]. In a study conducted in patients with diabetic nephropathy, the plasma concentration of this protein was reported to be significantly higher compared with healthy individuals. Thus, it was said to be a useful proinflammatory biomarker that could be used in the early diagnosis of diabetic nephropathy [37]. Similarly, another study including 87 patients with type 2 diabetes reported a significant increase in the serum concentration of chitinase‐3‐like protein 1 (YKL‐40), and a significant positive relationship was noted between this protein and NEFA and triglyceride concentrations [52]. Thus, previous studies [37, 51, 52] have shown that chitinase‐3‐like protein 1 affects carbohydrate and lipid metabolism in metabolic diseases such as diabetes and can be used in the early diagnosis of diabetes because it regulates the inflammatory response. Researchers have reported that inflammatory responses occur with an increase in BHBA concentration in cases of pregnancy toxemia [10, 12]. Therefore, the increase in chitinase‐3‐like protein 1 concentrations in goats with pregnancy toxemia in our study is supported by previous results found in diabetic patients [37, 52]. Chitinase‐3‐like protein 1 might be an effective new biomarker for the demonstration of inflammatory responses in goats with pregnancy toxemia. Furthermore, pregnancy toxemia and diabetes share a similar pathophysiology, and the decreased concentrations of this protein observed in people with diabetes in previous studies further support the potential of this marker as a diagnostic candidate in pregnancy toxemia.

Galectin‐1 has been reported to play a role in both the regulation of adipose tissue in humans and animals and the maintenance of homeostasis in adipose tissue in various metabolic diseases [32, 38, 53, 54]. However, in the field of veterinary medicine, few studies on galectin have been conducted despite increasing interest in this protein in human medicine in recent years. In a study involving patients with type 2 diabetes, it was found that the concentration of galectin‐1 differed significantly in patients compared with healthy individuals; its expression in adipocytes was increased [38]. In parallel, studies conducted by many different research groups have reported that a significant relationship exists between the concentration of galectin‐1 secreted from adipose tissue and metabolic diseases, and that galectin‐1 can serve as a useful biomarker [32, 38]. In our study, in accordance with previous reports [32, 38, 53], it was determined that galectin‐1 differed significantly in the pregnancy toxemia groups compared with the CG. The increase in the concentration of galectin‐1 in diabetes in humans and the promising results obtained in some therapeutic studies suggest that this protein could be used as a biomarker in the diagnosis and treatment of diabetes. In light of these previous studies on metabolism and diabetes in both humans and rats, the increased concentration of this protein in goats with pregnancy toxemia in our study suggests the potential of this protein as a biomarker for pregnancy toxemia in goats.

Our study had some limitations. The existence of differences between groups in some proteins not associated with pregnancy toxemia could not be explained. However, we did not aim to focus on the molecular pathways or mechanisms of the identified proteins. *C. hircus* (NCBI Taxonomy Number 9925, UniProtKB/Swiss‐Prot 258 proteins), commonly known as the domestic goat, is represented by approximately 75 488 protein sequences in the NCBI Protein database. Although that comprehensive collection is valuable, protein databases for *C. hircus* are quite limited compared to those for humans or model organisms such as mice and yeast, which negatively affects the efficiency of proteomic analyses for goats. This situation might lead to lower protein identification rates because the available reference sequences might not comprehensively cover all possible proteins present in a given sample. This problem hinders our full understanding of domestic goat biology and genetics and decreases the efficiency of research, such as that presented in our study. To address these challenges, it is essential to continuously update and expand the *C. hircus* protein database with new sequences and annotations. Expanding the *C. hircus* protein database provides a critical platform for more accurate biomarker discovery in pregnancy toxemia. As the database grows, it will support refined proteomic analyses that can identify stage‐specific protein signatures. These signatures have strong potential for translation into practical diagnostic tools, such as ELISA‐based tests or targeted mass spectrometry panels for early detection, disease monitoring, and prognosis in veterinary settings. In the longer term, such proteomic insights also could inform preventive herd health strategies and selective breeding programs aimed at improving metabolic resilience in pregnant goats. To do so, investments in international database projects should be increased, and interdisciplinary collaborations should be encouraged.

In conclusion, we determined that increased or decreased protein expressions in serum proteomics in pregnancy toxemia potentially could serve as biomarker candidates. In addition, when the findings obtained from our study are evaluated as a whole, it can be predicted that serum proteomic biomarkers can be determined using rapid tests that may facilitate effective diagnosis and treatment.

## Disclosure

The authors have nothing to report.

## Ethics Statement

This study was approved by the Bingöl University Animal Experiments Local Ethics Committee (Date: 2023/06; Decision No: 06/01). The authors declare human ethics approval was not needed.

## Conflicts of Interest

The authors declare no conflicts of interest.
